# Complete genome sequence of *Cronobacter muytjensii* bacteriophage vB_Cmu_VP5

**DOI:** 10.1128/mra.00271-26

**Published:** 2026-05-18

**Authors:** Peter Durovka, Andrej Minich, Tatiana Sedlackova, Michal Kajsik

**Affiliations:** 1 Medirex s.r.o., Bratislava, Slovakia; 2 Comenius University Science Park 685207 , Bratislava, Slovakia; Queens College Department of Biology, Queens, New York, USA

**Keywords:** *Cronobacter muytjensii*, bacteriophages, genome analysis

## Abstract

*Cronobacter muytjensii* is a gram-negative bacterium associated with public health concerns. Here, we report the characterization of cronophage vB_Cmu_VP5. Genome analysis indicates that vB_Cmu_VP5 is a member of the class Caudoviricetes. The genome contains 47 open reading frames, and zero tRNA genes, virulence factors, and antimicrobial resistance genes were predicted.

## ANNOUNCEMENT

The genus *Cronobacter* comprises opportunistic bacterial pathogens associated with infections in neonates, the elderly, and immunocompromised adults, including meningitis, necrotizing enterocolitis, and septicemia (1, 2). *Cronobacter muytjensii* is reported less frequently in clinical cases compared with other *Cronobacte*r spp. It is predominantly isolated from environmental sources (3). This announcement describes the process of isolating, sequencing, and annotating the genome of the bacteriophage vB_Cmu_VP5. Bacteriophage is capable of infecting and lysing *Cronobacter muytjensii* NTU895 isolated from infant formula in Brazil in 2012 (4). The phage vB_Cmu_VP5 was obtained by growth on *Cronobacter muytjensii* strain NTU895, which was cultivated in LB broth with filtered wastewater at 37°C overnight. A 50 mL wastewater sample was collected in 2020 from the intake pipe of a wastewater treatment plant in Modra, Slovakia (GPS: 48.32014539669544 N, 17.315005890767004 E). The sample was filtered using a 0.22 µm syringe filter, mixed with 50 mL of double-strength LB medium, and subsequently inoculated with bacteria at an initial concentration of 1 × 10³ CFU/mL. The phage was purified through three rounds of isolations from single plaques on double agar and amplified in liquid cultures. Further purification was achieved by subjecting the phage to ultracentrifugation in a cesium chloride gradient (5, 6).

Bacteriophage DNA was purified using a phage DNA isolation kit (NorgenBiotek, Canada). Next-generation sequencing libraries were prepared with a Nextera XT DNA library preparation kit, and phage was sequenced on an Illumina MiSeq platform (paired-end 150-bp reads) using 300-cycle v2 chemistry. FastQC (https://www.bioinformatics.babraham.ac.uk/projects/fastqc/) was used to control the quality of the 2,634,048 sequence reads, which were trimmed using Trimmomatic v0.40 and assembled using SPAdes v3.5.0 (7, 8). Assembly completeness was confirmed via CheckV v1.0.3 (9). The assembly resulted in a single contig with a length of 31,668 bp and, using 2,486,185 reads, a coverage of 9,800× was achieved. To ensure that the complete termini would be present, PCR products (forward, 5′-TGGCCTATGACATCACCGAG-3′; reverse, 5′-TTTTCAACCATTCCAGCCCG-3′) amplified from the contig ends were Sanger-sequenced. Protein-coding genes were predicted and annotated with a pipeline using Pharokka v1.8 (10), Phold v1.0.0 (11), and InterProScan v5.75-106.0 (12). The presence of tRNA was predicted using tRNAscan-SE v2.0.12 (13) and Aragorn v1.2.41 (14). The genomic terminus type was predicted with PhageTermVirome v4.3 (15). All analyses were conducted with default settings. Whole-genome comparisons were performed with average nucleotide identity (ANI) calculator (https://www.ezbiocloud.net/tools/ani) using the OrthoANIu algorithm (16). The phage life cycle was predicted with the PhageAI tool (17).

**Fig 1:**
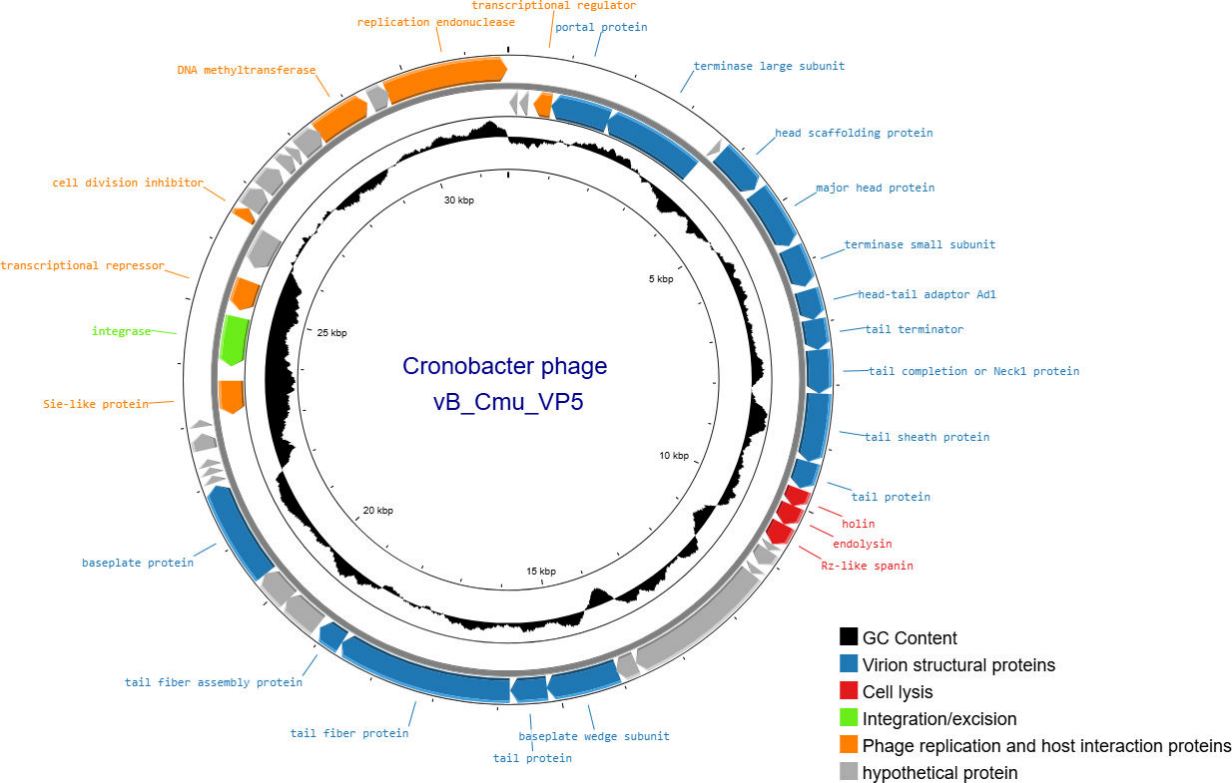
Annotation map of *Cronobacter* phage vB_Cmu_VP5, created using Proksee-Genome Analysis web server (19).

Bacteriophage vB_Cmu_VP5 was predicted to be temperate, with an estimated accuracy of 99.95%. It is able to infect and lyse *Cronobacter muytjensii* strain NTU895. vB_Cmu_VP5 has a 31,668-bp genome with a G+C content of 57.37%. A total of 47 open reading frames were annotated, with no detected tRNA genes, CRISPR elements, VFDB virulence factors, or CARD AMR genes, resulting in a protein-coding density of 94.97% (Fig. 1). Phage vB_Cmu_VP5 shares high nucleotide-level similarity with human metagenome *Caudoviricetes* sp. isolate cty6E2 (accession number BK032453) (18) with an ANI score of 95.77% and a genome coverage of 77.27% (Table 1).

**TABLE 1:** Genome characteristics and comparative analysis of vB_Cmu_VP5 with closest relative

Parameter	Result
*Cronobacter* phage vB_Cmu_VP5	
Genome size (bp)	31,668
Coverage (×)	9,800
GC content (%)	57.37
CheckV completeness (%)	100
CheckV quality	High
No. of CDS	47
No. of hypothetical proteins	22
No. of tRNAs	0
No. of antibiotic resistance genes	0
No. of bacterial virulence genes	0
Life cycle—Phage AI (%)	99.95—Temperate
Closest related phage MAG TPA_asm: bacteriophage sp. isolate cty6E2	
GenBank accession no.	BK032453.1
Taxonomy	Unclassified *Caudoviricetes*
Query coverage (%)	77.27
Percent identity (%)	95.77
Country	USA: Pittsburgh
Collection date	2013/2014

## Data Availability

The genome sequence of bacteriophage vB_Cmu_VP5 was deposited under GenBank accession number PX409136, BioProject accession number PRJNA922742, SRA accession number SRR35631953, and BioSample accession number SAMN51794128.
