# Erratum for Durovka et al., “Complete genome sequence of *Cronobacter muytjensii* bacteriophage vB_Cmu_VP5***”***

**DOI:** 10.1128/mra.00770-26

**Published:** 2026-07-30

**Authors:** Peter Durovka, Andrej Minich, Tatiana Sedlackova, Michal Kajsik

## ERRATUM

Volume 15, no. 6, e00271-26, 2026, https://doi.org/10.1128/mra.00271-26. Author Affiliations: The first listed affiliation should read “MediXbank, n.o., Nitra, Slovakia.”

